# Teleeducation: Pandemic Proofing Global Education

**DOI:** 10.3389/ijph.2022.1604872

**Published:** 2022-05-24

**Authors:** Janet Michel, Shiva Murugasampillay, Thomas C. Sauter, Marcel Tanner

**Affiliations:** ^1^ Department of Emergency Medicine, Inselspital, Bern University Hospital, Bern, Switzerland; ^2^ Global Public Heath Consultant, Geneva, Switzerland; ^3^ Swiss Tropical and Public Health Institute (Swiss TPH), Basel, Switzerland; ^4^ WHO, Public Health Services, University of Basel, Basel, Switzerland

**Keywords:** pandemic, decolonising global health, teleeducation, international collaborations, equitable access to global education

## Introduction

The COVID-19 pandemic has made mankind rethink travel, trade and globalization. Airspaces closed and video (Zoom, Microsoft teams) conferences and meetings became practical and cost-effective alternatives. Home office became the norm for many and education from primary schools to universities went online. We have termed this movement, the provision of education online as Teleeducation. The provision of some health care services went on line too, increasingly known as Telehealth (1). The arrival of MRNa vaccines brought renewed hope. The messages were, get your jabs and get your life back. Many people who could access vaccines got their jabs and when COVID passports were introduced, they got theirs and resumed travel, with no testing, no masks and no fear of contracting COVID-19 (2).

Summer in Europe brought relief as infections in Europe and the northern hemisphere fell (3), while at the same time the virus surged in the southern hemisphere. International travel re-opened and countries like Denmark declared COVID-19 as being over and the UK celebrated Freedom Day (3). In Oct–Nov 2021 infections started rising, in Austria, Netherlands, Belgium, Germany, United Kingdom, Switzerland and later France, Italy and Portugal (3). The discovery of the Omicron variant in November 2021 surprised the world once again. Many Countries closed borders and Netherlands and Austria went into new lock downs. Hospitals in Germany exceeded capacity and similar European Countries faced tough challenges of triaging care at times (4). The short-term sense of security brought about by the vaccines somewhat got eroded by the emergence of delta and omicron variants (5). Could this be a call for caution for the rest of the world to defer celebrating the end of the pandemic, to tread carefully and to keep watch on variant emergences and seasonal cycles?

Education has been affected by the pandemic and should not be postponed further. Besides economies, health and trade, education has been affected by the pandemic too. Education is the key to economic and social freedom, personal and professional development particularly in LMIC. Many students interrupted or postponed their education in 2020 and 2021. In a pandemic setting, many people get sick including students. The ability to continue learning while sick and or in quarantine, is an opportunity that became strengthened during the pandemic and could be capitalized on.

### Hurdles to Accessing Higher Education

Students from LMICs benefit from scholarships to study abroad. Some middle-class families from LMICs also sacrifice livelihoods to give an equitable start to the next generation in the globally competitive world, by sending them abroad to study. Travel restrictions have been put in place several times during this pandemic, interrupting education. Pre-departure tests and additional measures have been introduced including self-funded hotel quarantine on arrival and post arrival tests. All these are additional financial stumbling blocks for many students from LMICs (6).

## Time for a Rethink-Systems Thinking

**FIGURE 1:**
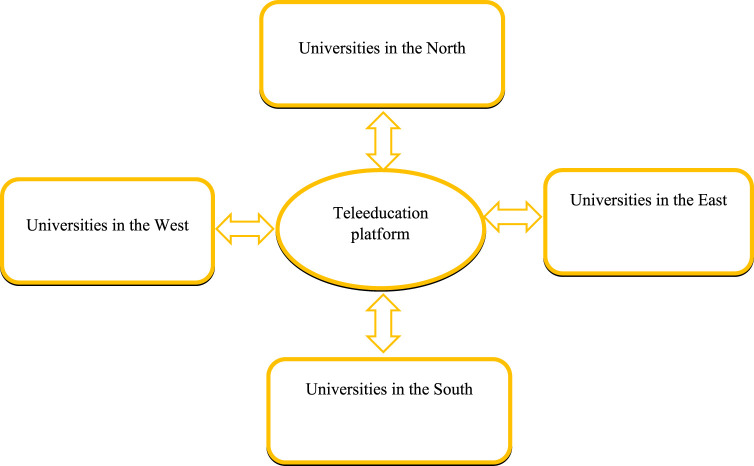
Teleeducation-a platform for equitable education for all (Teleeducation, Switzerland 2022).

The pandemic has indeed forced us to change our ways of thinking and doing things. In our view, a rethink in educational approaches is needed too. Pandemics are forecasted to become more frequent. Students the world over, need not interrupt or defer studies in pandemic contexts. The internet of things has brought the world closer than we ever imagined. Students can interact with teachers and other students all over the world from within the comfort of their own homes (7) or own home universities, without the need to travel-teleeducation. See Figure 1.

These initiatives would be in line with initiatives like decolonising global health, which are striving to redistribute funding, knowledge, authority, leadership and decision-making power among communities. Similarly, these initiatives could aid in reducing brain drain and ultimately strengthen health and educational institutions in LMICs (8, 9).

### Redirecting the High Budgets of Travel, Accommodation to Broad Band and Tech Infrastructure in LMIC Universities?

Systems thinking is a disciplined approach to examining problems accurately and comprehensively before acting. We believe systems thinking is called for to rethink International (North–South, East–West) higher education collaborations? Instead of hand picking a few students from LMICs and relocating them to the West to acquire higher education, why not take higher education to the LMICs, through collaborations and redirected broadband investments in Universities in the South and East. Not only do students from LMICs benefit, but lecturers from LMICs also get the opportunity to co-create global education and teach, research and publish in collaboration with universities from the west (7). The result is that more students from LMICs will access high quality education rather than a lucky few that get Scholarships or those with parents that can afford the western universities. These collaborations could enable lecturers in LMICs to teach courses albeit virtually, at universities in the west and lecturers in HICs to teach virtually at Universities in the LMICs. Virtual platforms can bring lecturers and students from LMIC, MIC and High Income Countries together, ensuring global equitable access to education (7).

### Conclusion

The COVID-19 pandemic is still here, evolving, a new variant, omicron has just been discovered, more mutations are expected, and the annual seasonal cycles are expected to continue, with variations in severity, depending on vaccine efficacy, coverage and levels of community immunity. New pandemics are expected in the decades to come. Is it not worth redirecting the high costs of travel to countries in the north (air, accommodation and relocation costs) to broadband and other infrastructural and institutional investments in LMIC universities, to build a teleeducation platform? We have seen how interconnected we are, and how a virus from one country can spread across the globe within a very short space of time. We believe systems thinking is called for. The pandemic is an opportune moment for higher education institutions to rethink and collaborate. These developments in our minds, fit into many initiatives including decolonising global health and decolonizing higher education and research, a move towards an equitable global and sustainable educational system-Teleeducation.

## References

[1] Field MJ. Introduction and Background. Washington (DC): National Academies Press (1996). Telemedicine: Institute of Medicine (US) Committee on Evaluating Clinical Applications of Telemedicine. Google Scholar

[2] UChicago Medicine. Vaccinated? How Your Life May Change after Getting the COVID-19 Vaccine (2021). Available at: https://www.uchicagomedicine.org/forefront/coronavirus-disease-covid-19/life-after-covid-vaccine (Accessed December 6, 2021). Google Scholar

[3] CNN. Denmark Ditched its Covid Rules Two Months Ago. Now It’s Joined Other EU Nations in Mulling New Restrictions (2021). Available at: https://www.cnn.com/2021/11/09/europe/denmark-restrictions-europe-covid (Accessed December 6, 2021). Google Scholar

[4] Al Jazeera. Vaccinated, Cured or Dead’: Germany Battles Surging COVID Cases | Coronavirus Pandemic Newse (2021). Available at: https://www.aljazeera.com/news/2021/11/22/austria-re-enters-covid-19-lockdown-as-cases-soar-anew-in-europe (Accessed December 6, 2021). Google Scholar

[5] CNN. Live Updates: Omicron Variant Spreads across the World (2021). Available at: https://edition.cnn.com/world/live-news/omicron-coronavirus-variant-12-06-21-intl (Accessed December 6, 2021). Google Scholar

[6] Gedela K. Are Current Systems of Global Health Academia Fit for Purpose? Lancet Glob Health (2021) 9:e1656. 10.1016/s2214-109x(21)00453-8 PubMed Abstract | 10.1016/s2214-109x(21)00453-8 | Google Scholar 34798024

[7] Frehywot S Vovides Y Talib Z Mikhail N Ross H Wohltjen H E-learning in Medical Education in Resource Constrained Low- and Middle-Income Countries. Hum Resour Health (2013) 11:4. 10.1186/1478-4491-11-4 PubMed Abstract | 10.1186/1478-4491-11-4 | Google Scholar 23379467 PMC3584907

[8] IHEID. How Do We Decolonise Global Health? Lessons from the Geneva Health Forum (2020). Available at: https://www.graduateinstitute.ch/communications/news/how-do-we-decolonise-global-health-lessons-geneva-health-forum (Accessed December 9, 2021). Google Scholar

[9] Rasheed MA. Navigating the Violent Process of Decolonisation in Global Health Research: a Guideline. Lancet Glob Health (2021) 9:e1640–e1641. 10.1016/s2214-109x(21)00440-x PubMed Abstract | 10.1016/s2214-109x(21)00440-x | Google Scholar 34798014

[10] Senge PM. The Fifth Discipline: The Art and Practice of the Learning Organization. London: Random House Business Books (1999). Google Scholar

